# Huntingtin Subcellular Localisation Is Regulated by Kinase Signalling Activity in the *StHdh^Q111^* Model of HD

**DOI:** 10.1371/journal.pone.0144864

**Published:** 2015-12-14

**Authors:** Kathryn R. Bowles, Simon P. Brooks, Stephen B. Dunnett, Lesley Jones

**Affiliations:** 1 Institute of Psychological Medicine and Clinical Neurosciences, MRC centre for Neuropsychiatric Genetics and Genomics, School of Medicine, Hadyn Ellis building, Maindy Road, Cardiff University, Cardiff CF24 4HQ, Wales, United Kingdom; 2 The Brain Repair Group, School of Biosciences, Cardiff University, Museum Avenue, Cardiff CF10 3AX, Wales, United Kingdom; Grenoble Institut des Neurosciences, Universite Grenoble Alpes, FRANCE

## Abstract

Huntington’s disease is a neurodegenerative disorder characterised primarily by motor abnormalities, and is caused by an expanded polyglutamine repeat in the huntingtin protein. Huntingtin dynamically shuttles between subcellular compartments, and the mutant huntingtin protein is mislocalised to cell nuclei, where it may interfere with nuclear functions, such as transcription. However, the mechanism by which mislocalisation of mutant huntingtin occurs is currently unknown. An immortalised embryonic striatal cell model of HD (*StHdh* ^*Q111*^) was stimulated with epidermal growth factor in order to determine whether the subcellular localisation of huntingtin is dependent on kinase signalling pathway activation. Aberrant phosphorylation of AKT and MEK signalling pathways was identified in cells carrying mutant huntingtin. Activity within these pathways was found to contribute to the regulation of huntingtin and mutant huntingtin localisation, as well as to the expression of immediate-early genes. We propose that altered kinase signalling is a phenotype of Huntington’s disease that occurs prior to cell death; specifically, that altered kinase signalling may influence huntingtin localisation, which in turn may impact upon nuclear processes such as transcriptional regulation. Aiming to restore the balance of activity between kinase signalling networks may therefore prove to be an effective approach to delaying Huntington’s disease symptom development and progression.

## Introduction

Huntington’s disease (HD) is an autosomal dominant neurodegenerative disorder caused by a CAG expansion within the first exon of the *HTT* gene, which gives rise to an expanded polyglutamine tract in the huntingtin protein. HD is primarily characterised by progressive motor abnormalities that manifest in the third to fourth decades of life, but is also commonly associated with cognitive impairments and psychiatric disturbances [1–3]. The caudate and putamen exhibit the most prominent cell loss [4]; GABAergic medium spiny neurons (MSNs) are the first to be affected, and is later accompanied by widespread atrophy of cortical structures [5]. Neuronal dysfunction occurs prior to both striatal atrophy and overt motor symptom onset [6,7], and therefore cell death and degeneration in HD-affected neuronal cells are likely to develop following an initial period of dysregulation of numerous cellular processes [8].

Patients with HD have been reported as having a low incidence of tumour formation, as well as a lower glucose metabolism [9,10]. As the activity of kinase signalling cascades are well characterised in the development of tumorigenesis [11] and the regulation of glucose metabolism [12], these peripheral characteristics of HD could be a result of huntingtin-mediated alterations in growth factor-responsive kinase pathways. Anti-apoptotic kinase signalling pathways in particular have been found to be important for prolonging neuronal survival in HD models by inhibiting cellular changes elicited by mutant huntingtin [13].

The most thoroughly characterised survival pathway in HD is the protein kinase B (AKT) pathway; enhanced activation of AKT in models of HD has been attributed to the reduced expression of its inhibitor, PH domain leucine-rich repeat protein phosphatase 1 (PHLPP1), which is suppressed in Hdh^Q111^, R6/1, R6/2 and HD94 striata, as well as in human putamen [14]. Aberrant interactions between mutant huntingtin and growth factor receptor bound protein 2 (GRB2) may also directly activate growth factor signalling cascades that are upstream of AKT activation, thus enhancing AKT phosphorylation [15]. Mitogen activated protein kinase kinase (MEK) has been less thoroughly investigated in the context of HD, and the neuroprotective effects of its activation remain under debate [16,17]. However, MEK1 activation has been found to enhance the phosphorylation of huntingtin [18], which can reduce mutant huntingtin toxicity [19–24].

Huntingtin has numerous potential cellular functions in both the cytoplasm and the nucleus; dynamic shuttling between these compartments is likely to be a mechanism by which a complex variety of huntingtin-associated activities can be regulated [25,26]. Within cell nuclei, huntingtin has been found to localise to nuclear structures such as promyelocytic leukaemia (PML) bodies and the nucleolus [27–29], associates with a variety of transcription factors, and is able to bind to DNA directly. These mechanisms can all be modified by the presence of an expanded polyglutamine repeat within mutant huntingtin [29–38]. These interactions implicate huntingtin as having a necessary role in transcription and RNA processing within the nucleus, as well as in the assembly of nuclear matrix bound protein complexes [29,39]. These observations support transcriptional dysregulation as an important mechanism contributing to HD pathogenesis. Subcellular mislocalisation of mutant huntingtin to cell nuclei may directly affect transcription: however, as AKT and MEK pathways are known to play a role in gene expression, either by direct regulation or through the phosphorylation of other proteins [16,40–43], disruption of these upstream kinase signalling pathways by mutant huntingtin may also be a mechanism by which transcriptional regulation is altered in HD.

By stimulating cells from the *StHdh* ^*Q111*^ immortalised embryonic striatal cell model of HD, which carries 111 CAG repeats [44], with epidermal growth factor (EGF), we have created a dynamic model that demonstrates that both AKT and MEK signalling pathways may contribute to the regulation of huntingtin subcellular localisation, as well as to the control of gene expression. We find that both AKT and MEK pathways are aberrantly regulated in cells carrying an expanded polyglutamine repeat, and inhibition of these pathways corrected mutant huntingtin mislocalisation and gene expression to a phenotype more closely resembling that of wild type cells. We suggest that aberrant control of kinase signalling may be a mechanism influencing mutant huntingtin mislocalisation, which in turn may alter transcriptional dysregulation, thus contributing to HD pathogenesis.

## Materials & Methods

### Immortalised Cell culture

The *StHdh* ^*Q111*^ immortalised embryonic striatal cell model used in this project was originally derived from primary striatal neurons cultured from the Hdh^Q111^ knock-in mouse model of HD [44]; as such, wild type cells (*StHdh* ^*Q7/7*^) carry two alleles each containing 7 CAG repeats, and heterozygote (*StHdh* ^*Q7/111*^) and homozygote (*StHdh* ^*Q111/111*^) cells carry either one or two mutant alleles carrying 111 CAG repeats, respectively. The *StHdh* ^*Q7/7*^, *StHdh* ^*Q7/111*^ and *StHdh* ^*Q111/111*^ cell lines utilised here were a kind gift from Marcy MacDonald (Molecular Neurogenetics Unit, Massachusetts General Hospital, Massachusetts, USA). Cells were grown and maintained in high glucose Dulbecco’s Modified Eagle Medium (DMEM) (Life Technologies) containing 1% Penicillin-Streptomycin solution (Sigma Aldrich), 1% 40mg/ml Geneticin (Life Technologies) and 10% fetal bovine serum (FBS) (PAA) in a humid environment at 33°C with 5% C02.

### Primary Cell Culture

Hdh^Q111^ mice were bred in house from a colony established from a stock supplied by Jackson laboratories for the specific purpose of this experiment. All experiments were conducted under the UK Animals (Scientific procedures) Act of 2015 and approved by local ethical review by Cardiff University Biological Standards Committee. Staged pregnant mice were sacrificed by cervical dislocation at embryonic day 14 (E14) in the absence of analgesics; an approved Schedule 1 method under the UK Animals (Scientific Procedures) Act 2015 at Cardiff University, School of Biosciences, within the JBIOS unit. Embryos were removed, the brains removed, and microdissection of the ganglionic eminence was carried out as previously described [45]. The resulting dissections were digested in 0.1% Trypsin (Lorne Laboratories) and cultured in DMEM/F12 containing 1% antibiotic-antimycotic solution, 2% B-27 supplement (all Life Technologies) and 10% FBS (Sigma-Aldrich). Cells were grown in a humid environment at 37°C with 5% C0_2_

### Antibodies

Ab109115 (Abcam) was used at a dilution of 1:100, and Mab2166 (Merck Millipore) was used at 1:1000 for immunofluorescence. S421 was a kind gift from Dr. Sandrine Humbert, (Institut Curie, Paris, France), and was used at 1:1000 for immunofluorescence. Anti-EGFR (New England Biolabs) was used at 1:50 for immunofluorescence and 1:1000 for western blots. Calreticulin (Abcam) was used at 1:500 for immunofluorescence, and α-tubulin was used at 1:5000 for western blots. Anti-CTIP2 (Abcam) was used at 1:500, and Anti-DARPP32 (Santa Cruz Biotechnology Inc.) was used at 1:200; both for immunofluorescence. Secondary antibodies; Goat-anti-Rabbit AlexaFluor 568 and Donkey-anti-Mouse AlexaFluor 488 (Life Technologies) were both used at 1:100 for immunofluorescence. Goat-anti-Rabbit AlexaFluor 680 (Life Technologies) and Goat-anti-mouse DyLight 800 were used for Western blots at 1:10,000 and 1:20,000, respectively.

### Immunofluorescence

Cells were fixed by incubation with 10% formalin solution (containing 4% paraformaldehyde; Sigma-Aldrich) for 15 min. at room temperature. Cells were permeabilised with 0.1% Triton x-100 (Life Technologies) and blocked with 1% (w/v) bovine serum albumin (BSA) (Life Technologies) before undergoing incubation with the appropriate antibodies. Cells were imaged using either a Leica SP5 confocal microscope, or a Leica DM6000B fluorescent microscope.

### Western blotting

Proteins were denatured and resolved on a 4–12% Bis-Tris gel, then electroblotted onto a PVDF membrane. Blots were blocked in 5% milk powder in PBS with 0.1% Tween (PBS-T) before incubation with the appropriate antibodies. Protein bands were visualised and quantified on a Licor® Odyssey® quantitative fluorescent imaging system.

### ELISA

Phospho-AKT1 (Ser473) and phospho-MEK1 (Ser217/221) PathScan® Sandwich ELISA kits (Cell Signalling Technology) were utilised to assay kinase activation and inhibition, and were carried out according to manufacturer’s instructions.

### Kinase activation and inhibition and huntingtin localisation

EGF (Life Technologies) was reconstituted and diluted to a stock solution of 100μg/ml in PBS and stored at -20°C. Cells were serum starved for 24 hours prior to EGF treatment, then incubated with 100ng/ml EGF in fresh serum free media for the appropriate length of time.

AKT inhibitor VIII (Merck Millipore) was reconstituted in dimethyl sulfoxide (DMSO) (Sigma Aldrich) to a stock solution of 0.1mM, and MEK 1/2 inhibitor (Merck Millipore) was initially reconstituted in DMSO, before being diluted to a 0.05mM stock solution in serum free media and stored at -20°C. Cells were serum starved for 24 hours prior to treatment with either kinase inhibitor. Each inhibitor was diluted to the appropriate concentration in serum free media, and was incubated with the cells for 2h prior to EGF stimulation. Serum free media containing the equivalent volume of DMSO alone was used as a control. EGF was diluted to 100ng/ml in fresh serum free media that also contained the appropriate concentration of each kinase inhibitor or DMSO before addition to the cells.

### RNA extraction

RNA was extracted from cells using phenol/chloroform, precipated in ethanol, and purified by silica membrane spin columns (RNeasy MinElute Cleanup kit (Qiagen)) according to manufacturer’s instructions. DNA contaminants were treated with TURBO™DNase (Life Technologies) according to manufacturer’s instructions.

### Quantitative RTPCR

1μg RNA was reverse transcribed using the High Capacity RNA-to-cDNA™ kit (Life Technologies), and the resulting cDNA was amplified using Sybr®Green mastermix (Life Technologies) containing the relevant oligonucleotide primer pair. The oligonucleotides used were as follows; *Egr1* forward: 5’- CCTATGAGCACCTGACCACA-3’ reverse: 5’- GGGATAACTCGTCTCCACCA-3’, *Arc* forward: 5’- GAGAGCTGAAAGGGTTGCAC-3’ reverse: 5’-ACGTAGCCGTCCAAGTTGTT-3’, *Ngfib* forward: 5’- CAATGCTTCGTGTCAGCACT-3’ reverse: 5’ TGGCGCTTTTCTGTACTGTG-3’. The thermal cycler protocol was as follows: *Stage 1*–95°C for 10 min. *Stage 2* (x40 cycles) - 95°C for 15 s, 55°C for 1 s. *Stage 3* (dissociation curve) - 95°C following a gradual ramp. All qRTPCR protocols were controlled and analysed by the Sequence Detection Systems (SDS) Version 2.3 programme by Life Technologies.

### Statistical analysis

Microscopy images were analysed blind to genotype, EGF stimulation and kinase inhibition by renaming image files to randomised numbers by individuals who were not involved in the project. Mean pixel intensities from nuclear, cytoplasmic and perinuclear regions of interest were measured from grayscale images using the GNU Image Manipulation Program 2.6.11, copyright 1995–2008 (S1 Fig). Every cell within each image was analysed; higher antibody binding elicited a higher signal. Nuclear/cytoplasmic (N/C) and nuclear/perinuclear (N/P) ratios were calculated for each cell individually in order to account for variation in background noise and to determine the presence of any alterations in huntingtin localisation between subcellular regions. All data was subject to analysis by the appropriate ANOVA, followed by post-hoc Tukey HSD tests.

## Results

### Epidermal growth factor receptor localisation in *StHdh* ^*Q111*^ cell lines

**Fig 1:**
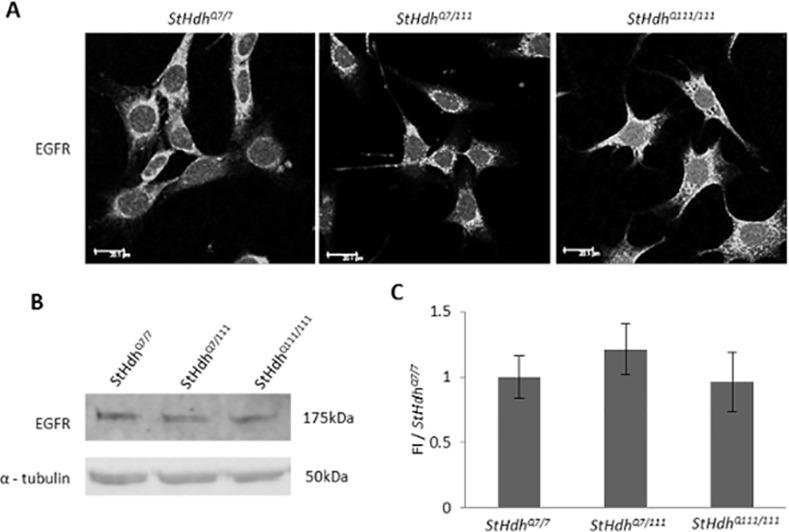
A. The EGFR is present in *StHdh* ^*Q7/7*^, *StHdh* ^*Q7/111*^ and *StHdh* ^*Q111/111*^, cells and has a similar subcellular localisation Scale bar = 20μm. B. Western Blot indicating similar expression of the EGFR protein in all three cell lines, α-tubulin was used as a loading control. C. Quantification of fluorescence intensity of protein bands in *B* normalised to loading control and expressed as a proportion of *StHdh* ^*Q7/7*^ (FI/*StHdh* ^*Q7/7*^); there are no significant differences in the levels of EGFR between *StHdh* ^*Q7/7*^, *StHdh* ^*Q7/111*^ and *StHdh* ^*Q111/111*^ cells. Error bars = ± SEM. Images are representative of multiple experiments. N = 3 replications.

To affirm the ability of the *StHdh* ^*Q111*^ cell lines to respond to EGF stimulation, the presence and localisation of the EGF receptor (EGFR) was investigated by immunofluorescence and western blotting (Fig 1). The EGFR was present in all three cell lines, and exhibited no significant differences in its expression or subcellular localisation between genotypes, which is consistent with previous observations in the same model [46].

### Differential kinase pathway activation in *StHdh* ^*Q111/111*^ cells

**Fig 2:**
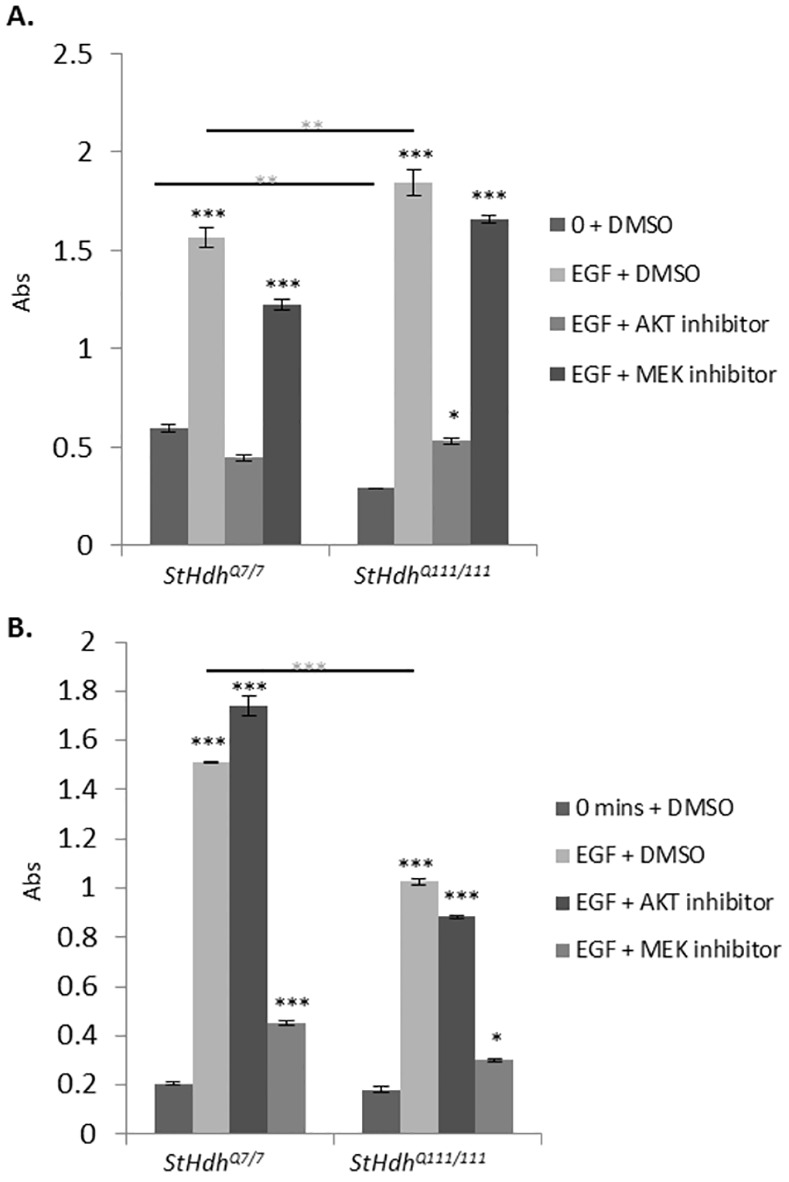
Mean absorbance (Abs) read at 450nm following a sandwich ELISA protocol for the detection of phosphorylated A. AKT1 and B. MEK1 in *StHdh* ^*Q7/7*^ and *StHdh* ^*Q111/111*^ cells at 0 mins and 10 mins of 100ng/ml EGF stimulation, either with or without a prior 2 hour incubation with 500nM AKT inhibitor VIII or 1μM MEK 1/2 inhibitor. In cases where inhibitors were not used, cells were incubated with the equivalent volume of DMSO for the same amount of time prior to treatment and processing. Error bars = ±SEM. Black asterisks denote a significant difference from 0 mins + DMSO. Grey asterisks indicate genotypic differences. N = 3 replications *p<0.05, ** p<0.01, *** p<0.001.

To determine whether kinase signalling pathways downstream of EGF stimulation show differential effects in the *StHdh* ^*Q111*^ cell model, we examined the phosphorylation of kinases AKT and MEK in *StHdh* ^*Q7/7*^ and *StHdh* ^*Q111/111*^ cells in response to EGF stimulation by ELISA assay (Fig 2).

There was a significant effect of both genotype (F_1, 8_ = 1044, p<0.001) and EGF stimulation (F_3, 8_ = 2504.74, p<0.001) on the level of AKT1 phosphorylated on serine 473 (S473) and MEK1 phosphorylated on serines 217/221 (S217/221). Post-hoc Tukey HSD analyses indicated that although *StHdh* ^*Q111/111*^ cells exhibited suppressed AKT1 phosphorylation compared to *StHdh* ^*Q7/7*^ cells at baseline (p<0.01), AKT1 phosphorylation was significantly increased in both genotypes following EGF stimulation (both p<0.001). However, when comparing the magnitude of phosphorylation following EGF stimulation, *StHdh* ^*Q111/111*^ cells showed an augmented response (p<0.05), thus resulting in a significantly increased level of phosphorylated AKT1 in *StHdh* ^*Q111/111*^ in comparison to *StHdh* ^*Q7/7*^ cells (p<0.01) (Fig 2).

While there was no difference in the level of phosphorylated MEK1 between genotypes at baseline, there was a strong phosphorylation response in both *StHdh* ^*Q7/7*^ (p<0.001) and *StHdh* ^*Q111/111*^ cells (p<0.001) following EGF stimulation. However, a suppressed response in *StHdh* ^*Q111/111*^ cells resulted in a significantly lower level of phosphorylated MEK1 in *StHdh* ^*Q111/111*^ cells following EGF stimulation than in *StHdh* ^*Q7/7*^ cells (p<0.001) (Fig 2).

### Inhibition of AKT and MEK phosphorylation

The efficacy and specificity of selective kinase inhibitors against AKT1 and MEK1 phosphorylation were tested in both *StHdh* ^*Q7/7*^ and *StHdh* ^*Q111/111*^ cells by ELISA assay (Fig 2). Treatment with AKT inhibitor VIII suppressed EGF-stimulated AKT1 phosphorylation in both cell lines, while having no significant effect on the phosphorylation of MEK1. Following AKT1 inhibition, there was no longer a significant difference in AKT1 phosphorylation between genotypes in response to EGF stimulation.

Treatment with MEK 1/2 inhibitor prior to EGF stimulation significantly suppressed the MEK1 phosphorylation response in both genotypes, but did not alter AKT1 phosphorylation. Despite significant inhibition of phosphorylation compared to EGF stimulation alone, MEK1 phosphorylation remained significantly higher than at baseline for both *StHdh* ^*Q7/7*^ cells (p<0.001) and *StHdh* ^*Q111/111*^ cells (p<0.05), and remained higher in *StHdh* ^*Q7/7*^ cells compared with *StHdh* ^*Q111/111*^ cells (p<0.01) (Fig 2).

### Alterations in huntingtin subcellular localisation in response to EGF stimulation

#### Amino-terminal huntingtin co-localises with endoplasmic reticulum

As kinase activation occurs upstream of huntingtin phosphorylation [47] and has been identified as a modulator of huntingtin nuclear localisation [21,22], we investigated whether EGF stimulation would alter the subcellular localisation of non-phosphorylated amino-terminal huntingtin epitopes. Prior experience with amino-terminal huntingtin antibodies within this lab has identified that they detect an isoform of huntingtin that localises in a region surrounding the nucleus. In order to better define this region for accurate quantification of immunofluorescence images, we looked at the co-localisation of amino-terminal huntingtin with the ER marker, calreticulin (S2 Fig). Calreticulin is present in all three cell lines in a region immediately surrounding the nucleus, and is not altered by the presence of mutant huntingtin. Furthermore, it co-localises with amino-terminal huntingtin and mutant huntingtin detected by Mab2166 (S2 Fig). We therefore defined the area in which HTT co-localises with calreticulin as the ‘perinuclear’ region for forthcoming experiments and image analyses (for further details of the analyses, refer to *Methods*).

#### EGF stimulation alters the localisation of amino-terminal huntingtin epitopes

**Fig 3:**
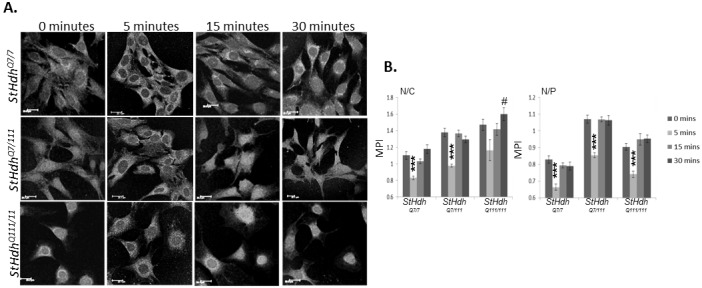
A. Subcellular localisation of an N-terminal epitope of huntingtin and mutant huntingtin in *StHdh* ^*Q7/7*^, *StHdh* ^*Q7/111*^ and *StHdh* ^*Q111/111*^ cell lines. Cells were fixed following 0, 5, 15 and 30 min. of stimulation with 100ng/ml EGF, labelled with Mab2166 against amino acids 181–810, then analysed by confocal microscopy. Scale bar = 20μm. B. Quantitative analysis of immunofluorescence images in *A*. Nuclear/Cytoplasmic (N/C) and Nuclear/Perinuclear (N/P) mean pixel intensity ratios (MPI) for *StHdh* ^*Q7/7*^, *StHdh* ^*Q7/111*^ and *StHdh* ^*Q111/111*^ cells following 0, 5, 15 and 30 min. of stimulation with 100ng/ml EGF. Mean pixel intensities were calculated from confocal microscopy images using GNU Image Manipulator. All images were randomised and analysed blind to genotype and length of time stimulated with EGF. Each condition consisted of 9 confocal microscopy images taken from 3 separate coverslips. n = 66–91. Error bars = ± SEM. Data representative of 3 experiments; * Denotes a significant difference from 0min.; # Denotes a significant difference from 5mins; */# p<0.05, **/## p<0.01, ***/### p<0.001.

Statistical analyses were focussed on the nuclear/cytoplasmic (N/C) and nuclear/perinuclear (N/P) ratios (refer to *Methods* for details), as these proved to be most informative and representative of the immunofluorescence images. There was a significant effect of genotype on the localisation of huntingtin as measured by the N/C ratio, when visualised using both Mab2166 (F_2, 847_ = 46.93, p<0.001) and Ab109115 (F_2, 856_ = 132.21, p<0.001; (Fig 3, S3 Fig), which was a result of an increase in the detection of nuclear huntingtin epitopes in *StHdh* ^*Q7/111*^ and *StHdh* ^*Q111/111*^ cells compared to *StHdh* ^*Q7/7*^ cells. A significant effect of genotype was also present in the analysis of the N/P ratio for both antibodies (Mab2166: F_2, 847_ = 134.48, p<0.001; Ab109115: F_2, 856_ = 228.6, p<0.001). Additionally, there was also a significant effect of EGF stimulation in both N/C and N/P ratios when using Mab2166 (N/C: F_3, 847_ = 26.43, p<0.001, N/P: F_3, 847_ = 49.2, p<0.001) and Ab109115 (N/C: F_3, 856_ = 6.08, p<0.001, N/P: F_3, 856_ = 18.64, P<0.001; Fig 3, S3 Fig). Post-hoc analyses indicate that this is due to a modest reduction in nuclear huntingtin in the N/P ratio for *StHdh* ^*Q7/7*^ cells following 15 mins of EGF stimulation (p<0.05) when detected by Ab109115, and after 5 mins when detected by Mab2166 in both N/C and N/P ratios (both p<0.05). Similarly, *StHdh* ^*Q7/111*^ cells exhibited a similar pattern, with a reduction in nuclear detection as measured by the N/P ratio from 15 mins of EGF stimulation when using Ab109115 (p<0.05) and in both the N/C and N/P ratios when using Mab2166 at 5 mins (both p<0.001). The reduced nuclear localisation following EGF stimulation when visualised using Ab109115 was abrogated in *StHdh* ^*Q111/111*^ cells, suggesting an impaired cellular response to EGF stimulation in the presence of mutant huntingtin. In contrast, there was a reduction in the N/P ratio alone at 5 mins in these cells when visualised using Mab2166 (p<0.001), indicating that the impairment may be huntingtin epitope-specific (Fig 3, S3 Fig).

#### Alterations in the subcellular localisation of amino-terminal huntingtin in response to EGF is independent of phosphorylation on serine 421

Although the phosphorylation of huntingtin on S421 has been shown to occur following AKT activation [48], which results in a neuroprotective reduction of nuclear huntingtin [22,47], we did not recognise any change in the subcellular localisation of huntingtin phosphorylated on S421 in these cells in response to EGF stimulation in any genotype (S4 Fig).

### Replicating the effect of EGF stimulation on huntingtin localisation in primary Hdh^Q111^ striatal cells

Primary cells were cultured from microdissected E14 Hdh^Q111^ mouse model striatum, from which the *StHdh* ^*Q111*^ cell model was originally derived [44]. In order to confirm the presence of striatal cells in primary cell cultures, a typical selection of cells was immunostained with antibodies against striatal cell markers dopamine- and cAMP-regulated neuronal phosphoprotein (DARPP-32) and CTIP2 (S5 Fig), both of which have been identified as being enriched within medium spiny neurons [49–51]. The majority of cells present in dissections were labelled with striatal markers, and therefore were considered to reflect the characterisation of an embryonic striatal cell population.

#### Confirming the presence of the EGFR in Hdh^Q111^ cells

**Fig 4:**
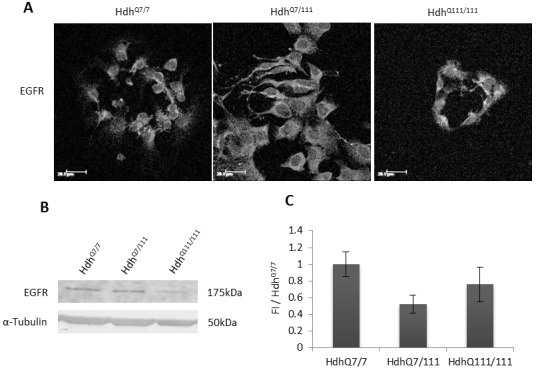
A. The EGFR is present in Hdh^Q7/7^, Hdh^Q7/111^ and Hdh^Q111/111^, cells and has a similar subcellular localisation. Scale bar = 20μm B. Western Blot indicating similar expression of the EGFR protein in all three genotypes, α-tubulin was used as a loading control. C. Quantification of fluorescence intensity of protein bands in *B* normalised to loading control and expressed as a proportion of Hdh^Q7/7^ (FI/Hdh^Q7/7^). Error bars = ± SEM. Images are representative of multiple experiments. N = 3 replications.

Immunofluorescence analysis revealed that the EGFR was present and had the same subcellular localisation in primary striatal cells from all three genotypes; Hdh^Q7/7^, Hdh^Q7/111^ and Hdh^Q111/111^ (Fig 4). There was a trend towards reduced levels of the EGFR in Hdh^Q7/111^ and Hdh^Q111/111^ cells as indicated by western blot, although this difference does not reach significance.

#### Kinase pathway activation in Hdh^Q111^ primary cells

**Fig 5:**
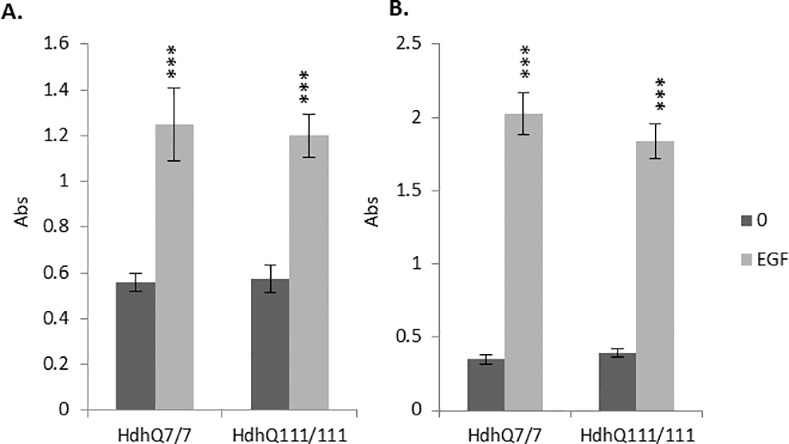
Mean absorbance (Abs) read at 450nm following a sandwich ELISA protocol for the detection of phosphorylated A. AKT1 and B. MEK1 in Hdh^Q7/7^ and Hdh^Q111/111^ primary cells at 0 mins and 10 mins of 100ng/ml EGF stimulation. Error bars = ±SEM. Black asterisks denote a significant difference from 0 mins + DMSO. N = 5 replications. *** p<0.001.

The phosphorylation of AKT1 and MEK1 following EGF stimulation was assayed in Hdh^Q111^ primary striatal cell cultures (Fig 5). Treatment of primary cultures with EGF significantly increased AKT1 and MEK1 phosphorylation in both Hdh^Q7/7^ and Hdh^Q111/111^ cells (all p<0.001), although in contrast to *StHdh* ^*Q111*^ cell lines, there was no observable difference between genotypes.

#### EGF stimulation alters the localisation of amino-terminal huntingtin epitopes in Hdh^Q111^ primary cells

**Fig 6:**
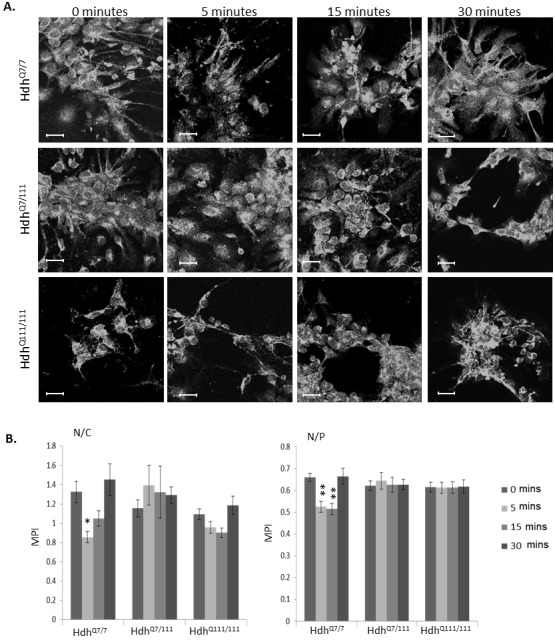
Subcellular localisation of an N-terminal epitope of huntingtin and mutant huntingtin in Hdh^Q7/7^, Hdh^Q7/111^ and Hdh^Q111/111^ primary cell lines. Cells were fixed following 0, 5, 15 and 30 min. of stimulation with 100ng/ml EGF, labelled with Mab2166, then analysed by confocal microscopy. Scale bar = 20μm. **B**. Quantitative analysis of immunofluorescence images in ***A*.** Nuclear/Cytoplasmic (N/C) and Nuclear/Perinuclear (N/P) mean pixel intensity ratios (MPI) for Hdh^Q7/7^, Hdh^Q7/111^ and Hdh^Q111/111^ primary cells following 0, 5, 15 and 30 min. of stimulation with 100ng/ml EGF. Mean pixel intensities were calculated from confocal microscopy images using GNU Image Manipulator. All images were randomised and analysed blind to genotype and length of time stimulated with EGF. Each condition consisted of 9 confocal microscopy images taken from 3 separate coverslips. n = 49–70. Error bars = ± SEM. Data representative of 3 experiments; * Denotes a significant difference from 0min.; *p<0.05, ** p<0.01.

The localisation of the huntingtin epitope detected by Mab2166 was primarily perinuclear in Hdh^Q7/7^, Hdh^Q7/111^ and Hdh^Q111/111^ cells, as observed in immortalised striatal cell lines (Fig 6). Analysis of mean pixel intensity ratios indicated a significant reduction in the N/C ratio in Hdh^Q7/7^ cells after 5 mins of EGF stimulation (p<0.05), and in the N/P ratio after both 5 and 15 mins (both p<0.01) of EGF stimulation. In contrast, there was no significant effect of EGF stimulation for either mutant huntingtin-carrying genotypes at any time point. There was a similar pattern of effect when cells were immunostained with Ab109115, although this was only mildly significant in the N/P ratio for Hdh^Q7/7^ cells (p<0.05; S6 Fig).

### Inhibition of AKT1 phosphorylation alters EGF-regulated huntingtin localisation in *StHdh* ^*Q111*^ cell lines

**Fig 7:**
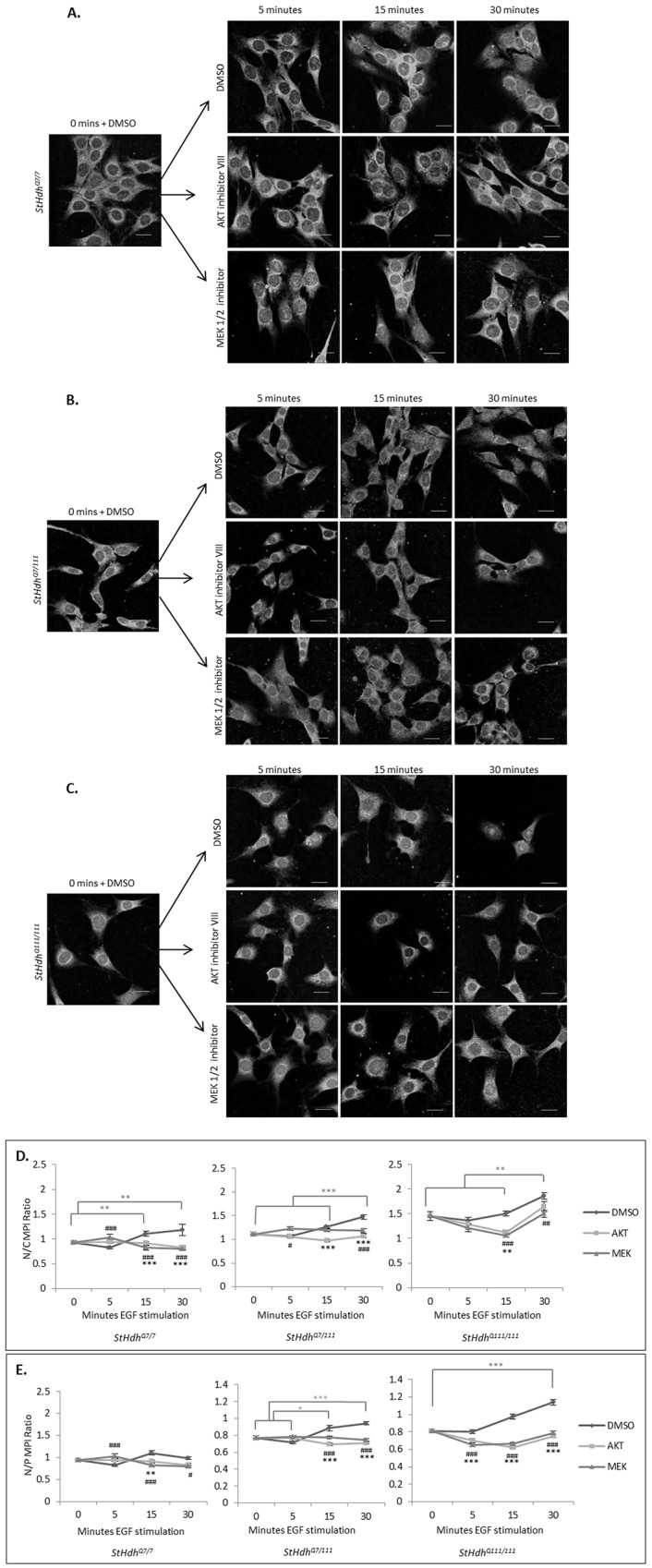
A. *StHdh* ^*Q7/7*^, B. *StHdh* ^*Q7/111*^ and C. *StHdh* ^*Q111/111*^ cells treated with either AKT inhibitor VIII, MEK 1/2 inhibitor, or the equivalent volume of DMSO for 2 hours prior to 0, 5, 15 and 30 mins stimulation with 100ng/ml EGF, then probed with amino-terminal huntingtin antibody Mab2166. Scale bar = 20μm. D-E. Quantification of mean pixel intensity (MPI) from images represented in *A-C* for the D. Nuclear/Cytoplasmic (N/C) ratio and E. Nuclear/Perinuclear (N/P) ratio. Error bars = SEM. Light grey bars and asterisks signify statistically significant differences between DMSO conditions. Black asterisks and hashes indicate statistically significant differences between DMSO vs AKT inhibitor conditions and DMSO vs MEK inhibitor conditions, respectively. Data representative of three experiments. n = 85–135. */# p<0.05, **/## p<0.01, ***/### p<0.001.

As EGF stimulation elicited AKT1 phosphorylation and altered the subcellular localisation of amino-terminal huntingtin in both *StHdh* ^*Q111*^ and primary Hdh^Q111^ cell lines, we sought to investigate whether the phosphorylation of AKT1 may be a mechanism that regulates huntingtin subcellular localisation. The subcellular localisation experiment was repeated, with the addition of a 2 hour incubation period with AKT VIII inhibitor, or the equivalent volume of DMSO, prior to stimulation with EGF (Fig 7, S7 Fig). Mab2166 and Ab109115 were again used to detect the localisation of amino-terminal huntingtin, and images were analysed in the same manner. There was a significant interaction between genotype and treatment condition for both N/C and N/P ratios (N/C F_18, 2785_ = 10.34, p<0.001; N/P F_18, 2785_ = 17.24, p<0.001), therefore suggesting a differential effect of AKT inhibition between genotypes (Fig 7, S7 Fig).

#### AKT1 inhibition alters the apparent localisation of huntingtin in *StHdh* ^*Q7/7*^ cells in an epitope-dependent manner

A significant reduction in nuclear huntingtin was not replicated in *StHdh* ^*Q7/7*^ cells following EGF stimulation when detected by Mab2166, which may be due to the presence of DMSO (Fig 7). However, there was still a trend towards reduced nuclear huntingtin detection at 5 mins of stimulation in both N/C and N/P ratios, and the original observation was replicated when Ab109115 was used for detection (S7 Fig). AKT1 inhibition had no significant effect at the 5 minute time point following EGF stimulation in either Ab109115 or Mab2166 immunostained cells. However, AKT1 inhibition did appear to prevent increased nuclear Mab2166 detection of huntingtin at 15 and 30 mins, which was likely elicited by DMSO (Fig 7, both p<0.01). In contrast, there was no effect of AKT1 inhibition on huntingtin localisation when detected by Ab109115.

#### AKT1 inhibition reduces the nuclear detection of huntingtin in *StHdh* ^*Q7/111*^ and *StHdh* ^*Q111/111*^ cells

Similar to the effect of EGF stimulation on the subcellular localisation of huntingtin in *Sthdh* ^*Q7/7*^ cells in the presence of DMSO, a large reduction in nuclear huntingtin detection by Mab2166 at 5 mins of EGF stimulation was not fully replicated in either *StHdh* ^*Q7/111*^ or *StHdh* ^*Q111/111*^ cells when DMSO was present, although both lines exhibited a trend towards this effect. In both cell lines there was an increase in nuclear huntingtin with increased length of EGF stimulation, which is observable in both N/C and N/P ratios (Fig 7, S7 Fig). This effect could be visualised using either Mab2166 or Ab109115 antibodies, and may be a result of DMSO-induced toxicity. Inhibition of AKT1 counteracts this effect; in *StHdh* ^*Q7/111*^ cells, there is a significant reduction in both N/C (p<0.001) and N/P (p<0.001) ratios from 15 mins of EGF stimulation when AKT1 is inhibited. In *StHdh* ^*Q111/111*^ cells, the same nuclear reduction is apparent from 5 mins in the N/P ratio (p<0.001) and from 15 mins in the N/C ratio (p<0.01; Fig 7). A similar result is produced when visualised with Ab109115; nuclear detection of huntingtin is significantly reduced from 5 mins in both *StHdh* ^*Q7/111*^ (N/C p<0.05) and *StHdh* ^*Q111/111*^ (N/C p<0.05) cells, although this effect is considerably more marked in *StHdh* ^*Q111/111*^ cells, and persists for the entirety of the experiment (S7 Fig).

### MEK inhibition alters huntingtin localisation in a similar manner to AKT inhibition

Inhibition of MEK1 was carried out in parallel with the AKT1 inhibition experiment, and therefore shared the same DMSO controls. MEK1 inhibition had a strikingly similar effect on the nuclear presence of huntingtin in response to EGF stimulation as AKT1 inhibition did in all three genotypes (Fig 7, S7 Fig). *StHdh* ^*Q7/7*^ cells exhibited increased nuclear huntingtin detection in the N/C (p<0.001) and N/P (p<0.001) ratios following 5 mins of EGF stimulation when MEK1 was inhibited, as detected by Mab2166 (Fig 7). This effect was also observed when detected by Ab109115 (S7 Fig).

Similar to the effect of AKT1 inhibition in *StHdh* ^*Q7/111*^ cells, there was a modest effect of MEK1 inhibition on huntingtin localisation when detected by both Mab2166 and Ab109115; there was a reduction in the N/P ratio at 15 mins and 30 mins (both p<0.001) when visualised with Mab2166 (Fig 7), and a modest reduction in the N/C ratio at 15 mins (p<0.05) when visualised with Ab109115 (S7 Fig).

*StHdh* ^*Q111/111*^ cells showed a similar pattern of huntingtin localisation change following MEK1 inhibition as following AKT1 inhibition (Fig 7, S7 Fig). There was a substantial reduction in the detection of nuclear huntingtin from 5 mins post- EGF stimulation following MEK1 inhibition, which was observed primarily in the N/P ratio (all time points p<0.001) when using Mab2166 (Fig 7). This effect was particularly strong when Ab109115 immunostaining was analysed, with both N/C and N/P ratios being significantly lower when MEK1 was inhibited at all time points (all p<0.001; S7 Fig).

### Dysregulation of immediate early gene expression is downstream of AKT and MEK signalling

**Fig 8:**
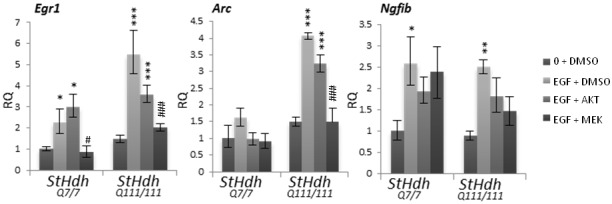
Relative quantitation (RQ) values representing gene expression fold change of *Egr1*, *Arc* and *Ngfib* in *StHdh* ^*Q7/7*^ and *StHdh* ^*Q111/111*^ cells following inhibition with either 500nM AKT inhibitor VIII, 1μM MEK 1/2 inhibitor or the equivalent volume of DMSO, followed by 0 or 2 hours of 100ng/ml EGF stimulation. Statistical analysis was conducted on ΔC_t_ values. Error bars = ± SEM. Asterisks denote a significant difference from 0 + DMSO, hashes indicate a significant difference from EGF + DMSO. Data representative of two experiments. N = 6.*/# p<0.05, **/## p<0.01, ***/### p<0.001.

The regulation of huntingtin localisation has been implicated as a mechanism for transcriptional control [19,23,25,26,52]. We therefore compared the expression of three immediate early (IE) genes downstream of the EGF signalling pathway between *StHdh* ^*Q7/7*^ and *StHdh* ^*Q111/111*^ cells, as these exhibited the most disparate huntingtin localisation responses to EGF stimulation and kinase inhibition. The expression of *Egr1*, *Arc* and *Ngfib* were measured by qRTPCR in response to EGF stimulation alone, and following either AKT1 or MEK1 inhibition. The transcriptional responses of these genes were typically more reactive in *StHdh* ^*Q111/111*^ cells, which elicited larger gene expression fold changes in response to EGF stimulation than in *StHdh* ^*Q7/7*^ cells (Fig 8). EGF stimulation significantly increased the expression of *Egr1* in both genotypes (F_3, 24_ = 17.95, p<0.001) although this effect was augmented in *StHdh* ^*Q111/111*^ cells (fold change x5.5, p<0.001) compared to *StHdh* ^*Q7/7*^ cells (fold change x2.24, p<0.05). This was an effect that was also observed in primary Hdh^Q7/7^ and Hdh^Q111/111^ cells (S8 Fig). AKT1 inhibition had no effect on *Egr1* expression in *StHdh* ^*Q7/7*^ cells, and although there was a trend towards a reduction in *Egr1* expression in *StHdh* ^*Q111/111*^ cells, this did not reach significance. In contrast, MEK1 inhibition suppressed the effect of EGF stimulation on *Egr1* expression in both genotypes (*StHdh* ^*Q7/7*^ p<0.05, *StHdh* ^*Q111/111*^ p<0.001; Fig 8).

The expression of *Arc* exhibited a similar pattern; while there was a larger effect of EGF in *StHdh* ^*Q111/111*^ cells (fold change x4, p<0.001) compared to *StHdh* ^*Q7/7*^ cells (fold change x1.6, n.s.), AKT1 inhibition had no effect on *Arc* expression in either genotype. By contrast, MEK1 inhibition prevented the large increase in *Arc* expression observed in *StHdh* ^*Q111/111*^ cells following EGF stimulation (p<0.001). The expression of *Ngfib* increased to a similar extent in both genotypes following EGF stimulation (F_3, 24_ = 8.07, p<0.001. *StHdh* ^*Q111/111*^ fold change x2.5, *StHdh* ^*Q7/7*^ fold change x2.59, both p<0.05); this effect was partially suppressed by both AKT1 and MEK1 inhibition for both *StHdh* ^*Q7/7*^ and *StHdh* ^*Q111/111*^ cells, but did not reach significance (Fig 8).

## Discussion

**Fig 9:**
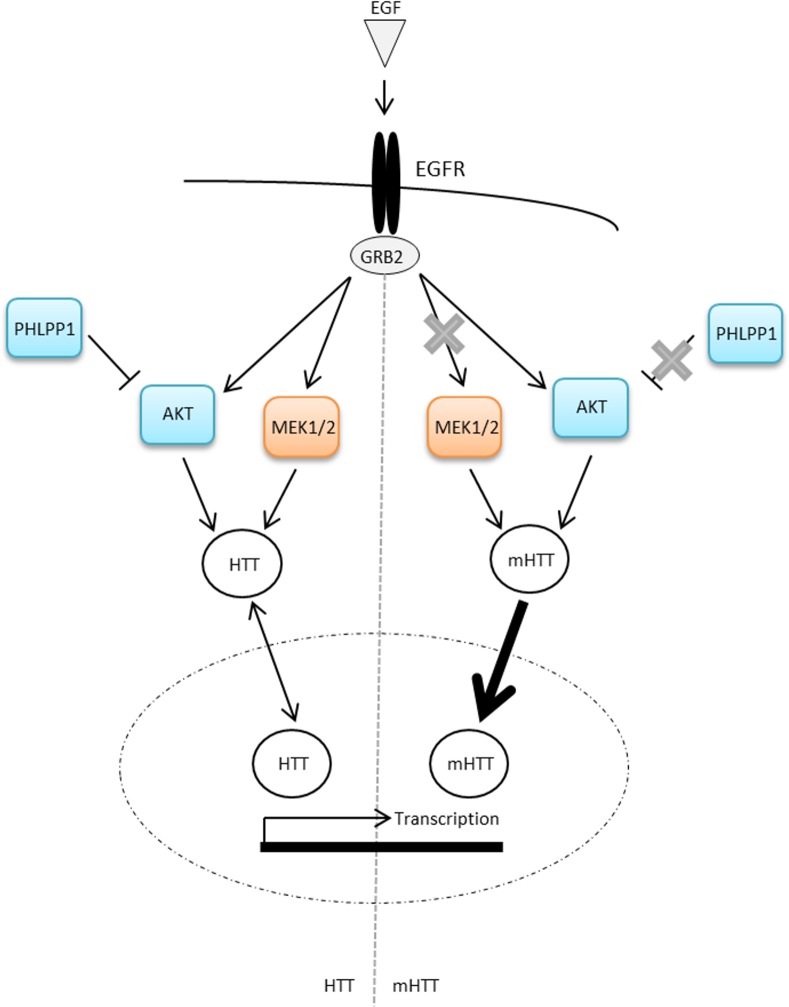
Simplified cartoon of the hypothesised relationship between EGF-stimulated kinase activation, huntingtin localisation and transcriptional regulation. Upon binding to the EGFR, EGF elicits the phosphorylation of MEK and AKT. In the presence of mutant huntingtin, aberrant interactions with GRB2 and suppressed PHLPP1 expression serve to alter the phosphorylation response. In turn, active MEK and AKT regulate the subcellular localisation of huntingtin, possibly by post-translational modification; however this regulation is impaired due to mutant huntingtin-associated aberrant kinase phosphorylation. Regulation of huntingtin nuclear localisation may then influence transcriptional control via multiple mechanisms. As such, altered nuclear localisation of mutant huntingtin could disrupt the control of these mechanisms and would result in altered transcriptional regulation.

We demonstrate that EGF stimulation elicits differential effects on the activation of the MEK1 and AKT1 kinase signalling pathways downstream of the EGF receptor in *StHdh* ^*Q7/7*^ and *StHdh* ^*Q111/111*^ cell lines, despite similar expression levels and localisation of the EGFR. We also demonstrate that dysregulation of these pathways may alter the subcellular localisation of huntingtin in both *StHdh* ^*Q111*^ and Hdh^Q111^ cell lines, which may impact upon downstream transcriptional control (Fig 9).

Baseline levels of AKT are relatively low in adult brain [53], however its activation rises following cellular stress and injury, and it invokes a protective response [54]. AKT exerts its protective effects by phosphorylating and inactivating components of the cell death machinery, such as B-cell lymphoma-2 associated death promoter (BAD), caspase-9, forkhead box proteins (FOXOs) and inhibitor of kappa-β kinase (IKK) [48,55]. Enhanced AKT activity has previously been identified in the *StHdh* ^*Q111/111*^ cell line [13,23,42], as well as in a neural stem cell model of HD [56], which may reflect a neuroprotective compensatory response in order to counteract the excitotoxic effects of mutant huntingtin [13,54]. For example, increasing AKT activity in a *Drosophila* model of HD reduced neurotoxicity [57], and reduced the number of intranuclear inclusions formed in a striatal cell model expressing mutant huntingtin [48].

Less information is currently available regarding the role of MEK1 signalling in models of HD, although its activation has previously been found to be reduced in *StHdh* ^*Q111*^ cells [46]. Consistent with Ginés et al. [13], we have identified suppressed MEK1 phosphorylation in *StHdh* ^*Q111/111*^ cells following EGF stimulation. There is evidence that huntingtin may be phosphorylated by MEK1 activation [18], although the implications of this mechanism are yet to be elucidated. A constitutively active form of MEK1 was found to be protective against cell death in a truncated-mutant huntingtin expressing PC12 model of HD [16], although by contrast, the induction of MEK1 activity provided no protection against polyglutamine induced lethality in a polyglutamine-overexpressing *Drosophila* model [17]. ERK1/2 activity has been found to be neuroprotective in models of HD [16,58] and as it lies downstream of MEK1 activation, it may be inferred that its action is representative of MEK1 activity.

However, we did not observe differential phosphorylation responses to EGF stimulation in primary Hdh^Q111^ cell cultures. This could be because mutant huntingtin requires a longer period of time to accumulate the underlying level of dysregulation responsible for these differential effects to be observed. Alternatively, the differential phosphorylation response may be model specific; multiple opposing effects of mutant huntingtin presence on kinase signalling pathways have previously been reported that vary depending on the model being investigated [59]. Regardless of the lack of a differential genotype effect on AKT1 and MEK1 phosphorylation in primary cells, EGF stimulation still altered huntingtin localisation and gene expression in Hdh^Q111^ cells in a manner similar to that observed in *StHdh* ^*Q111*^ cell lines, which could suggest that the extent of kinase phosphorylation may not be the most instructive indicator of kinase pathway integrity.

As kinase activation and inhibition has previously been associated with the regulation of the subcellular localisation of huntingtin [21–23], we investigated whether the differential responses in AKT1 and MEK1 phosphorylation following EGF stimulation could be reflected in the subcellular localisation of huntingtin. We demonstrate that EGF stimulation elicits differential apparent subcellular localisation of huntingtin and mutant huntingtin amino-terminal epitopes in both *StHdh* ^*Q111*^ and primary Hdh^Q111^ cells, and suggest that endogenous kinase signalling pathways may contribute to the regulation of either huntingtin localisation or conformation (Fig 9).

The neuroprotective effects of AKT activity have been proposed to be exerted through its phosphorylation of huntingtin on S421 [48,55], which has been found to reduce the toxicity of mutant huntingtin and the nuclear accumulation of toxic mutant huntingtin fragments, as well as restoring axonal transport [22,57,60–62]. However, we did not identify any changes in the localisation of huntingtin phosphorylated on S421 in response to EGF stimulation, which may be a result of the experimental design; other studies have elicited huntingtin S421 phosphorylation by the overexpression of constitutively active AKT or by insulin-like growth factor (IGF-1) stimulation [48,60,63], or have investigated the effects of phospho-mimetic mutations of huntingtin S421 [22], whereas the phosphorylation of endogenous huntingtin by EGF stimulation has not previously been assessed.

While other post-translational modifications may be contributing to the regulation of huntingtin subcellular localisation, which were not assayed here [21,23,24,64–68], it has not been established whether an alteration in the absolute localisation of huntingtin is observed following EGF stimulation, or whether there has been a change in conformation that would result in the exposure of alternative epitopes undetectable by the antibodies used in this study. While our results are consistent between both Mab2166 and Ab109115 antibodies, some variation in huntingtin detection was observed; this may be due to differences in binding affinity to specific huntingtin conformations. However, using both Mab2166 and Ab109115 we observe that the presence of nuclear huntingtin is increased in the context of the expanded polyglutamine; a phenotype previously described in this cell model [23,25,44,64] and in other models of HD [27,29,69–74].

Stimulation with EGF reduced the nuclear pool of huntingtin in *StHdh* ^*Q7/7*^ and Hdh^Q7/7^ cells, although this effect was apparent primarily between the nucleus and the region of the cell we defined as ‘perinuclear,’ which is consistent with the observation that huntingtin accumulates in the perinuclear region prior to nuclear translocation [75]. Huntingtin is also known to localise to the Golgi body and late endosomes [76,77], so increased localisation in the perinuclear region following stimulation may be representative of a role for huntingtin in the recycling and degradation of the EGFR, as has been established in human fibroblasts [78].

We also demonstrate that the effect of EGF stimulation on the localisation of mutant huntingtin is suppressed in *StHdh* ^*Q111/111*^ and Hdh^Q111/111^ cells; this suppression is consistent with the inhibited kinase phosphorylation and gene expression responses to EGF stimulation in other cell and *Drosophila* models of HD [79,80], as well as with the delayed EGFR degradation and altered trafficking that has been observed in human HD fibroblasts [78]. However, as we observe a trend towards a reduction of the EGFR in Hdh^Q7/111^ and Hdh^Q111/111^ cells, it is possible that this may also be contributing to the suppressed response in these cells.

The nuclear translocation of huntingtin has been previously proposed as a mechanism for regulating its function [25,26]. Multiple mechanisms may explain the ability of huntingtin to shuttle between the nucleus and cytoplasm[24,26,64,66,81,82]. The association of huntingtin with exogenous chaperones to traverse the nuclear pore, and the availability of nuclear export/nuclear localisation signals (NES/NLS) on huntingtin itself may change with alterations in its conformation [26,83–85], and are therefore potential mechanisms by which EGF stimulation could be altering the localisation of huntingtin. However, the process that may be regulating huntingtin localisation in our current model requires clarification.

In order to determine whether the dysregulation of AKT1 and MEK1 phosphorylation observed in *StHdh* ^*Q111/111*^ cells could be responsible for altered mutant subcellular localisation, we repeated the experiment whilst inhibiting either AKT1 or MEK1 activation in response to EGF stimulation. Inhibition of either kinase elicited an increase in nuclear huntingtin in *StHdh* ^*Q7/7*^ cells, indicating that the subcellular localisation of huntingtin can be regulated by AKT1 and MEK1 activity. The increase in nuclear huntingtin is not as marked when visualised with Mab2166 compared to Ab109115, which may be due to detection of an alternate epitope of huntingtin, or variation in antibody specificity. Alternatively, differences in subcellular localisation following EGF stimulation may be due to their ability to detect different huntingtin cleavage products. While both antibodies detect amino-terminal huntingtin, multiple aspartyl protease cleavage sites lie between the huntingtin binding sites for each antibody [86]; the resulting huntingtin fragments would therefore only be detectable by Ab109115, and would be able to more readily shuttle between cellular compartments by passive diffusion [19]. With continued EGF stimulation, this initial nuclear accumulation is followed by a slight reduction in nuclear huntingtin detection with both Ab109115 and Mab2166. Both AKT1 and MEK1 inhibition elicited similar effects on huntingtin localisation, and are therefore likely to be affecting huntingtin in a similar manner. However, whether this may be due to a shared downstream kinase or to similar, but distinct, mechanisms of action remains unclear and requires additional investigation.

Compared to the effect observed in *StHdh* ^*Q111/111*^ cells, the result of kinase inhibition in *StHdh* ^*Q7/7*^ cells was very modest. AKT1 and MEK1 inhibition in *StHdh* ^*Q111/111*^ cells elicited a dramatic reduction in nuclear mutant huntingtin following treatment with EGF. The expanded polyglutamine may therefore be modifying the normal interactions between huntingtin and elements downstream of kinase activation, which consequently has an effect on mutant huntingtin subcellular localisation. A reduction in nuclear huntingtin was observed in *StHdh* ^*Q7/7*^ cells following EGF stimulation alone, which may suggest that when aberrant activation of the MEK1 and AKT1 pathways is prevented, the *StHdh* ^*Q111/111*^ cellular phenotype becomes more similar to that of *StHdh* ^*Q7/7*^ cells. Increased nuclear localisation of huntingtin is typically considered to be pathogenic, and is associated with transcriptional dysregulation [87,88]; therefore reducing the presence of nuclear mutant huntingtin by inhibiting aberrant kinase signalling may prove to be beneficial for the appropriate regulation of cellular processes. To our knowledge, the manipulation of specific kinase signalling pathways has not yet been carried out in murine models of HD *in vivo* in order to determine any potential effects of kinase phosphorylation on phenotype and survival.

The shuttling of huntingtin between cellular compartments is associated with the regulation of its function [25,26], including the control of gene expression [19,23,37], so alterations in nuclear huntingtin localisation could be a regulatory mechanism for the aberrant transcriptional control that has been identified in *StHdh* ^*Q111/111*^ cells and other models of HD [89–91]. We therefore decided to investigate whether EGF stimulation and kinase inhibition altered the expression of a panel of IE genes downstream of the EGF pathway [40,41]. The extent of IE gene expression changes following EGF stimulation was typically larger in *StHdh* ^*Q111/111*^ cells, which is consistent with the AKT hyperactivity also observed in *StHdh* ^*Q111/111*^ cells. However, the integrity of the MEK1 signalling pathway appears to be more relevant for the control of the expression of *Egr1*, *Arc* and *Ngfib*, as its inhibition had a much more substantial effect on IE gene expression than the inhibition of AKT1.

We propose that kinase signalling is capable of modulating huntingtin localisation in both immortalised *StHdh* ^*Q111*^ and primary Hdh^Q111^ cell models of HD. In particular, MEK1 and AKT1 signalling are able to regulate huntingtin localisation and transcriptional control in the *StHdh* ^*Q111*^ cell model of HD, although the mechanism by which these processes may be associated remains unknown (Fig 9). MEK1 activity could be more relevant for transcriptional control in the *StHdh* ^*Q111*^ cell model of HD than AKT1 due to the greater impact of its inhibition on IE expression. However, as MEK1 phosphorylation was suppressed in *StHdh* ^*Q111/111*^ cells, and both AKT1 and MEK1 inhibition are able to alter huntingtin localisation in a similar manner, it is possible that a synergistic mechanism between multiple dysregulated pathways contributes to aberrant transcriptional control and huntingtin mislocalisation in models of HD. Alternatively, as similar huntingtin localisation and *Egr1* expression was observed in Hdh^Q111^ primary cells in the absence of differential MEK1 phosphorylation, it may be possible the extent of MEK1 phosphorylation does not accurately reflect downstream activity that is more directly impacting huntingtin localisation and transcriptional regulation. It also remains unclear from our model whether nuclear mutant huntingtin is having a direct effect upon transcriptional regulation, or whether another mechanism downstream of AKT1 and MEK1 signalling pathways is impacting upon transcriptional regulation in parallel with the regulation of huntingtin localisation.

As there is substantial overlap and shared targets between multiple kinase signalling pathways, it may be beneficial to target the restoration of the balance between pro- and anti-apoptotic mechanisms in HD rather than any one pathway in isolation. Despite being a neuroprotective anti-apoptotic pathway, the counter-intuitive inhibition of a hyper-activated AKT pathway may prove to be beneficial by restoring normal huntingtin localisation and IE gene expression by redressing the balance between pro- and anti-apoptotic pathways. However, suppressing AKT alone could ultimately enhance mutant huntingtin toxicity by failing to counter pro-apoptotic mechanisms. Therefore, a multi-target approach to restore the balance of compensatory and synergistic signal transduction mechanisms may be an efficacious therapeutic approach; this may be achieved by targeting upstream modulators of multiple kinase pathways, or by manipulating several targets simultaneously.

We demonstrate that the mislocalisation of mutant huntingtin is an early phenotype present in both an immortalised, and primary, embryonic cell model of HD, which can be modulated by the stimulation of kinase signalling pathways downstream of the EGFR. In particular, dysregulation of AKT1 and MEK1 kinase signalling pathways may contribute to aberrant huntingtin subcellular localisation. We hypothesise that the regulation of huntingtin localisation by kinase signalling could be a mechanism associated with transcriptional control, as the inhibition of both AKT1 and MEK1 activation was associated with alterations in both huntingtin localisation and IE gene expression. However, this study does not elucidate a direct functional relationship between huntingtin localisation and gene expression, and it is likely that mutant huntingtin impacts multiple mechanisms within cell nuclei. While we replicate the effect of EGF stimulation on the localisation of huntingtin in primary cells derived from the Hdh^Q111^ mouse model of HD, our findings require additional validation in multiple models of HD before firm conclusions about the relationship between kinase signalling, huntingtin localisation and IE gene expression can be drawn. We have also not yet clarified the mechanism by which the alterations in huntingtin subcellular localisation have occurred. However, we suggest that the presence of an expanded huntingtin polyglutamine repeat may be responsible for alterations in kinase signalling that subsequently leads to the mislocalisation of mutant huntingtin, which may in turn contribute to the aberrant control of gene expression observed in HD. By using embryonic cell models of HD, we demonstrate that cellular dysfunction is likely to be present throughout development, which may have implications for the understanding of HD developmental pathogenesis. The inability of these mechanisms to maintain survival-associated functions with cumulative mutant huntingtin insult may then lead to the observable pathogenic disease cascade. Therefore the investigation of early signalling dysfunction in HD models may provide an efficacious approach to developing novel therapeutic targets to delay age of onset and slow symptom progression.

## Supporting Information

S1 Fig An example of immunofluorescence image quantification using GNU Image Manipulator. For every cell, a circular region was individually selected within the nucleus (N), ‘perinuclear’ region (P) and cytoplasm (C), and a histogram displaying the mean pixel intensity for the selected region was generated. Within each cell, N/C and N/P pixel intensity ratios were calculated in order to compare the relative localisation of the huntingtin immunostaining, and to control for background variance between images and coverslips. Cell populations to be imaged were located by DAPI nuclear staining to avoid investigator bias. For each experiment, mean pixel intensities for N, P and C regions were measured blind to genotype and condition, and were taken from all cells present within 9 microscopy images derived from 3 individual coverslips. The presented data is a result of multiple experiments.
(TIF)

S2 Fig A-B. The ER marker Calreticulin was used to delineate the area designated as ‘perinuclear’ in these analyses. The amino-terminal epitope of huntingtin detected by Mab2166 colocalises with the ER marker calreticulin in the region immediately surrounding cell nuclei in *StHdh* ^*Q7/7*^, *StHdh* ^*Q7/111*^ and *StHdh* ^*Q111/111*^ cells. This localisation pattern of huntingtin was then categorised as ‘perinuclear’ in following experiments. ‘Cytoplasmic’ localisation was considered as away from the more densely localised huntingtin in the ‘perinuclear’ area, which would not colocalise with calreticulin. **B**. 4x magnification of images in ***A***. Magenta = huntingtin, green = calreticulin. Scale bar = 20μm.
(TIF)

S3 Fig Subcellular localisation of an N-terminal epitope of huntingtin and mutant huntingtin in *StHdh* ^*Q7/7*^, *StHdh* ^*Q7/111*^ and *StHdh* ^*Q111/111*^ cell lines. Cells were fixed following 0, 5, 15 and 30 min. of stimulation with 100ng/ml EGF, labelled with Ab109115 against amino acids 1–100 of huntingtin, then analysed by confocal microscopy. Scale bar = 20μm. **B**. Quantitative analysis of immunofluorescence images in *A*. Nuclear/Cytoplasmic (N/C) and Nuclear/Perinuclear (N/P) mean pixel intensity ratios (MPI) for *StHdh* ^*Q7/7*^, *StHdh* ^*Q7/111*^ and *StHdh* ^*Q111/111*^ cells following 0, 5, 15 and 30 min. of stimulation with 100ng/ml EGF. Mean pixel intensities were calculated from confocal microscopy images using GNU Image Manipulator. All images were randomised and analysed blind to genotype and length of time stimulated with EGF. Each condition consisted of 9 confocal microscopy images taken from 3 separate coverslips. Error bars = ± SEM. Data representative of 3 experiments. n = 60–87; * Denotes a significant difference from 0min.; # Denotes a significant difference from 5mins; */# p<0.05, **/## p<0.01, ***/### p<0.001.
(TIF)

S4 Fig Subcellular localisation of an N-terminal epitope of huntingtin and mutant huntingtin phosphorylated on S421 in *StHdh* ^*Q7/7*^, *StHdh* ^*Q7/111*^ and *StHdh* ^*Q111/111*^ cell lines. Cells were fixed following 0, 5, 15 and 30 min. of stimulation with 100ng/ml EGF, labelled with anti-S421, then visualised by fluorescence microscopy. Each condition consisted of 9 confocal microscopy images taken from 3 separate coverslips. Scale bar = 20μm.
(TIF)

S5 Fig A representative image demonstrating the proportion of DARRP-32 and CTIP2 positive cells in primary cultures from Hdh^Q111^ mice. 891 cells were assayed for these striatal cell markers, of which 93.83% were positively labelled.
(TIF)

S6 Fig A. Subcellular localisation of an N-terminal epitope of huntingtin and mutant huntingtin in Hdh^Q7/7^, Hdh^Q7/111^ and Hdh^Q111/111^ primary cell lines. Cells were fixed following 0, 5, 15 and 30 min. of stimulation with 100ng/ml EGF, labelled with Ab109115, then analysed by confocal microscopy. Scale bar = 20μm. B. Quantitative analysis of immunofluorescence images in *A*. Nuclear/Cytoplasmic (N/C) and Nuclear/Perinuclear (N/P) mean pixel intensity ratios (MPI) for Hdh^Q7/7^, Hdh^Q7/111^ and Hdh^Q111/111^ primary cells following 0, 5, 15 and 30 min. of stimulation with 100ng/ml EGF. Mean pixel intensities were calculated from confocal microscopy images using GNU Image Manipulator. All images were randomised and analysed blind to genotype and length of time stimulated with EGF. Each condition consisted of 9 confocal microscopy images taken from 3 separate coverslips. n = 49–70. Error bars = ± SEM. Data representative of 3 experiments; * Denotes a significant difference from 0min.; *p<0.05, ** p<0.01.
(TIF)

S7 Fig A. *StHdh* ^*Q7/7*^, B. *StHdh* ^*Q7/111*^ and C. *StHdh* ^*Q111/111*^ cells treated with either AKT inhibitor VIII, MEK 1/2 inhibitor, or the equivalent volume of DMSO for 2 hours prior to 0, 5, 15 and 30 mins stimulation with 100ng/ml EGF, then probed with amino-terminal huntingtin antibody Ab109115. Scale bar = 20μm. D-E. Quantification of mean pixel intensity (MPI) from images represented in *A-C* for the D. Nuclear/Cytoplasmic (N/C) ratio and E. Nuclear/Perinuclear (N/P) ratio. Error bars = SEM. Light grey bars and asterisks signify statistically significant differences between DMSO conditions. Black asterisks and hashes indicate statistically significant differences between DMSO vs AKT inhibitor conditions and DMSO vs MEK inhibitor conditions, respectively. Data representative of three experiments. n = 78–140. */# p<0.05, **/## p<0.01, ***/### p<0.001.
(TIF)

S8 Fig Relative quantitation (RQ) values representing gene expression fold change of *Egr1* in Hdh^Q7/7^ and Hdh^Q111/111^ primary cells following stimulation for 0 or 2 hours with 100ng/ml EGF stimulation. Statistical analysis was conducted on ΔC_t_ values. *Egr1* expression in both genotypes was significantly increased following EGF stimulation (both p<0.001), and the extent of this increase was significantly larger in Hdh^Q111/111^ primary cells compared to Hdh^Q7/7^ cells (p<0.05). Error bars = ± SEM. N = 5. * p<0.05, ** p<0.01, ***p<0.001.
(TIF)
