# Correction: Huntingtin Subcellular Localisation Is Regulated by Kinase Signalling Activity in the *StHdh^Q111^* Model of HD

**DOI:** 10.1371/journal.pone.0165069

**Published:** 2016-10-13

**Authors:** Kathryn R. Bowles, Simon P. Brooks, Alis C. Hughes, Stephen B. Dunnett, Lesley Jones

Dr. Alis C. Hughes should be included in the author byline. Dr. Hughes should be listed as the third author, and with the affiliation 1: Institute of Psychological Medicine and Clinical Neurosciences, MRC centre for Neuropsychiatric Genetics and Genomics, School of Medicine, Hadyn Ellis building, Maindy Road, Cardiff University, Cardiff CF24 4HQ, Wales, United Kingdom. The contributions of this author are as follows: Conceived and designed the experiments, wrote the manuscript.

The correct citation is: Bowles KR, Brooks SP, Hughes AC, Dunnett SB, Jones L (2015) Huntingtin Subcellular Localisation Is Regulated by Kinase Signalling Activity in the *StHdh*^*Q11*^1 Model of HD. PLoS ONE 10(12): e0144864. doi:10.1371/journal.pone.0144864
