# Supplementary figures and images for: Huntingtin Subcellular Localisation Is Regulated by Kinase Signalling Activity in the StHdhQ111 Model of HD
Source: PLoS One. 2015 Dec 14;10(12):e0144864. doi: 10.1371/journal.pone.0144864 (PMC4679340; doi:10.1371/journal.pone.0144864)

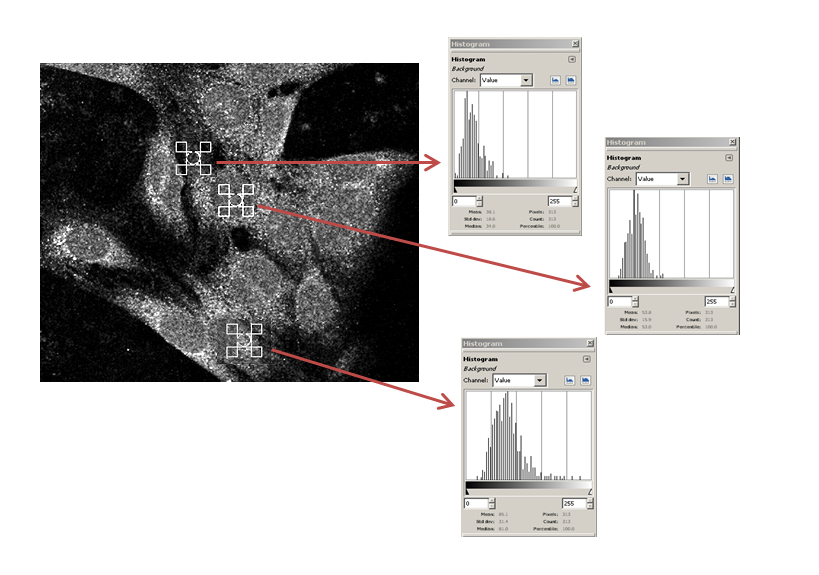

Supplement: S1 Fig — For every cell, a circular region was individually selected within the nucleus (N), ‘perinuclear’ region (P) and cytoplasm (C), and a histogram displaying the mean pixel intensity for the selected region was generated. Within each cell, N/C and N/P pixel intensity ratios were calculated in order to compare the relative localisation of the huntingtin immunostaining, and to control for background variance between images and coverslips. Cell populations to be imaged were located by DAPI nuclear staining to avoid investigator bias. For each experiment, mean pixel intensities for N, P and C regions were measured blind to genotype and condition, and were taken from all cells present within 9 microscopy images derived from 3 individual coverslips. The presented data is a result of multiple experiments. (TIF) [file pone.0144864.s001.tif]

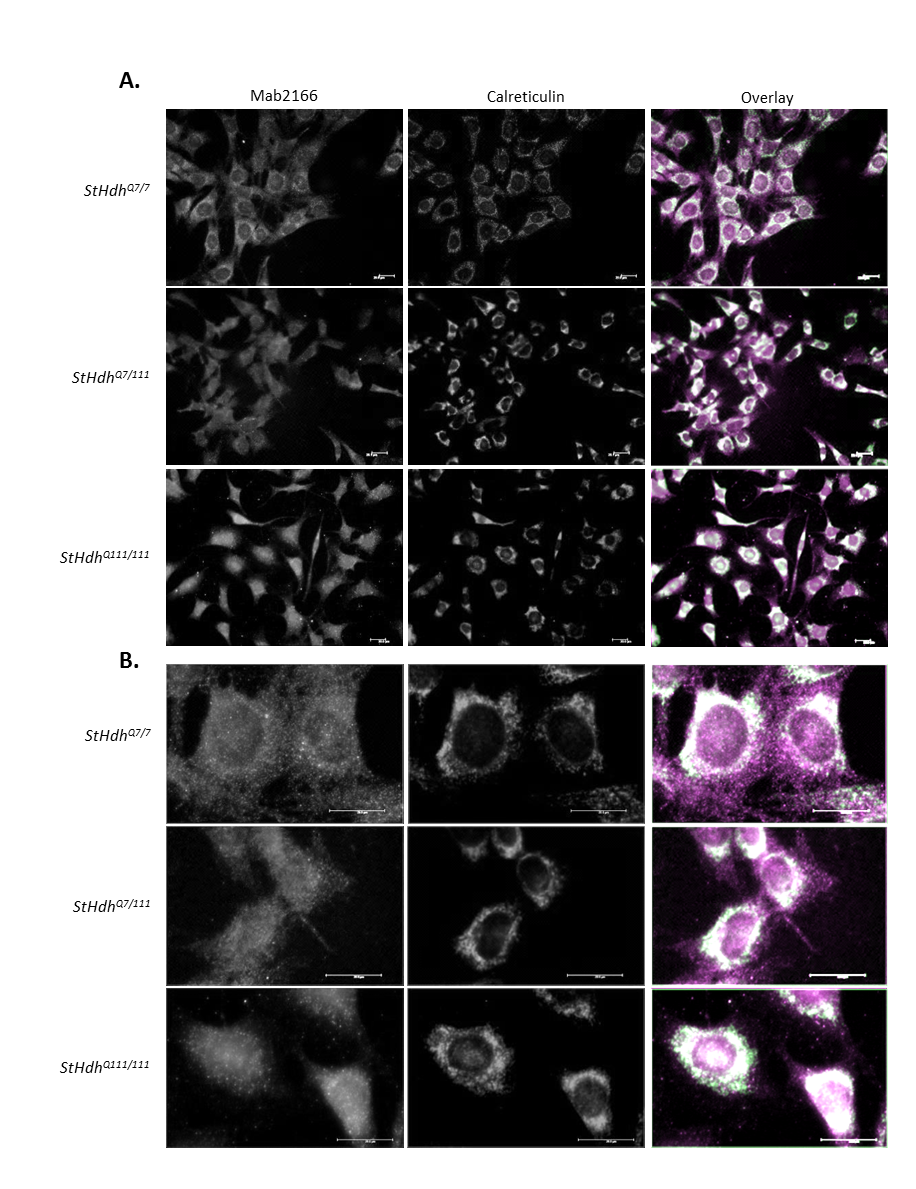

Supplement: S2 Fig — The amino-terminal epitope of huntingtin detected by Mab2166 colocalises with the ER marker calreticulin in the region immediately surrounding cell nuclei in StHdh Q7/7, StHdh Q7/111 and StHdh Q111/111 cells. This localisation pattern of huntingtin was then categorised as ‘perinuclear’ in following experiments. ‘Cytoplasmic’ localisation was considered as away from the more densely localised huntingtin in the ‘perinuclear’ area, which would not colocalise with calreticulin. B. 4x magnification of images in A. Magenta = huntingtin, green = calreticulin. Scale bar = 20μm. (TIF) [file pone.0144864.s002.tif]

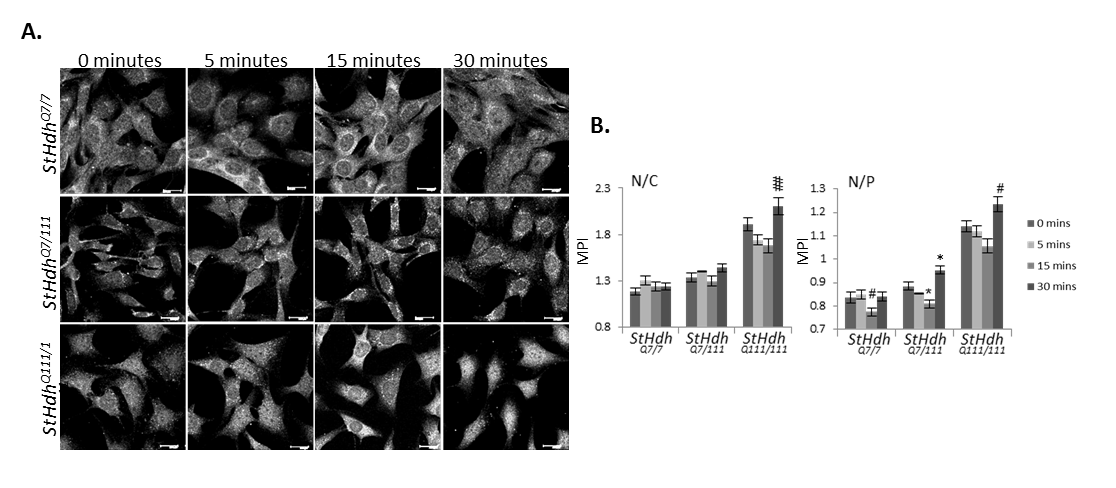

Supplement: S3 Fig — Cells were fixed following 0, 5, 15 and 30 min. of stimulation with 100ng/ml EGF, labelled with Ab109115 against amino acids 1–100 of huntingtin, then analysed by confocal microscopy. Scale bar = 20μm. B. Quantitative analysis of immunofluorescence images in A. Nuclear/Cytoplasmic (N/C) and Nuclear/Perinuclear (N/P) mean pixel intensity ratios (MPI) for StHdh Q7/7, StHdh Q7/111 and StHdh Q111/111 cells following 0, 5, 15 and 30 min. of stimulation with 100ng/ml EGF. Mean pixel intensities were calculated from confocal microscopy images using GNU Image Manipulator. All images were randomised and analysed blind to genotype and length of time stimulated with EGF. Each condition consisted of 9 confocal microscopy images taken from 3 separate coverslips. Error bars = ± SEM. Data representative of 3 experiments. n = 60–87; * Denotes a significant difference from 0min.; # Denotes a significant difference from 5mins; */# p<0.05, **/## p<0.01, ***/### p<0.001. (TIF) [file pone.0144864.s003.tif]

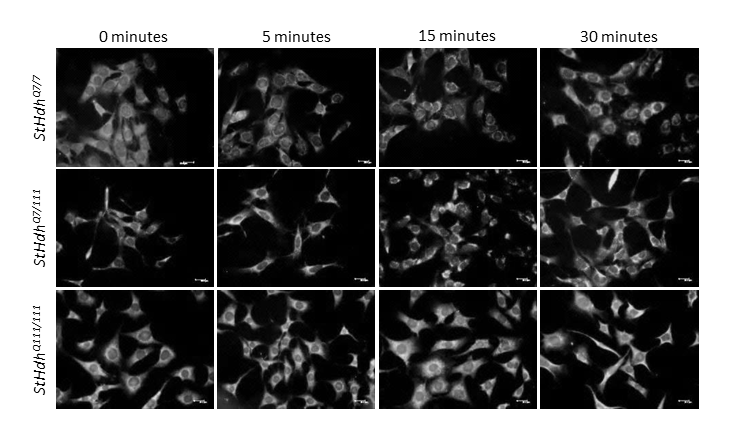

Supplement: S4 Fig — Cells were fixed following 0, 5, 15 and 30 min. of stimulation with 100ng/ml EGF, labelled with anti-S421, then visualised by fluorescence microscopy. Each condition consisted of 9 confocal microscopy images taken from 3 separate coverslips. Scale bar = 20μm. (TIF) [file pone.0144864.s004.tif]

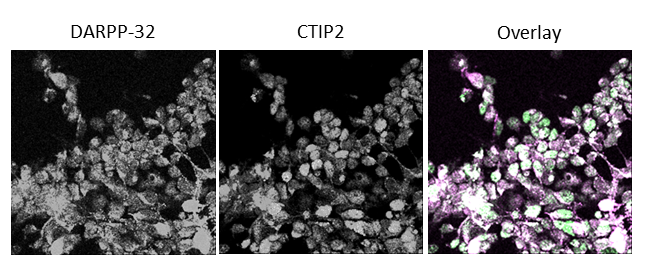

Supplement: S5 Fig — 891 cells were assayed for these striatal cell markers, of which 93.83% were positively labelled. (TIF) [file pone.0144864.s005.tif]

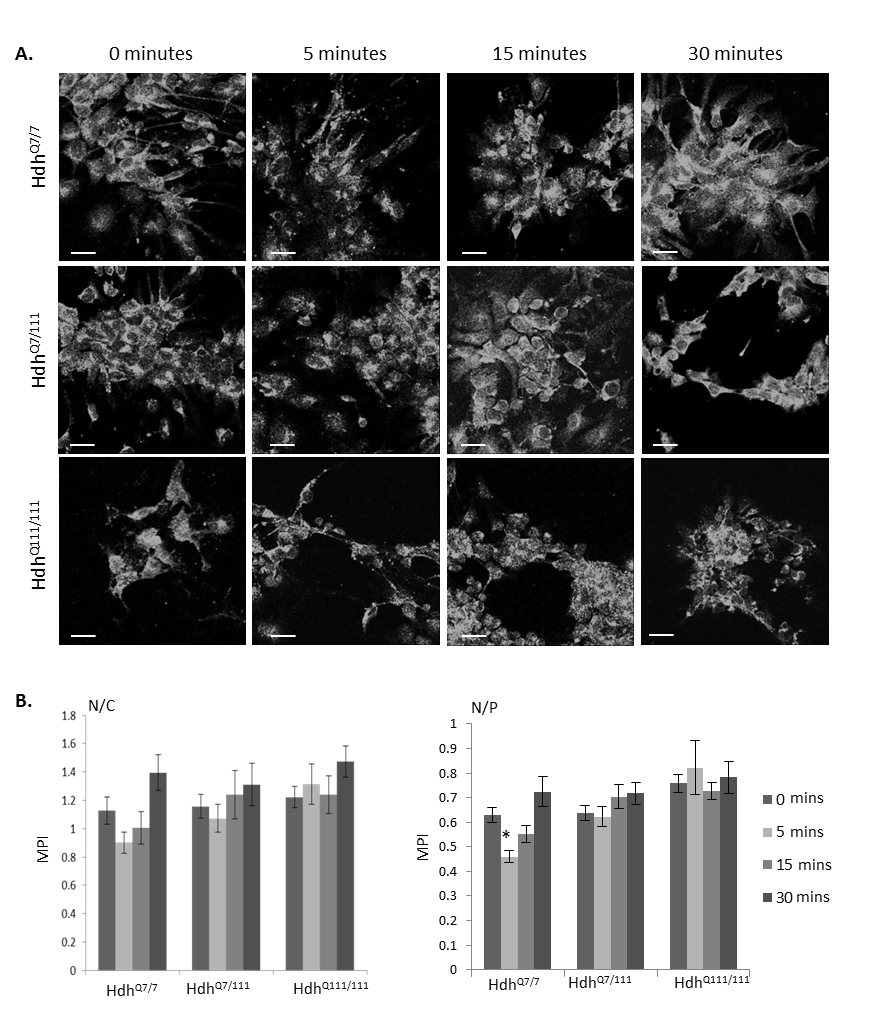

Supplement: S6 Fig — Mean pixel intensities were calculated from confocal microscopy images using GNU Image Manipulator. All images were randomised and analysed blind to genotype and length of time stimulated with EGF. Each condition consisted of 9 confocal microscopy images taken from 3 separate coverslips. n = 49–70. Error bars = ± SEM. Data representative of 3 experiments; * Denotes a significant difference from 0min.; *p<0.05, ** p<0.01. (TIF) [file pone.0144864.s006.tif]

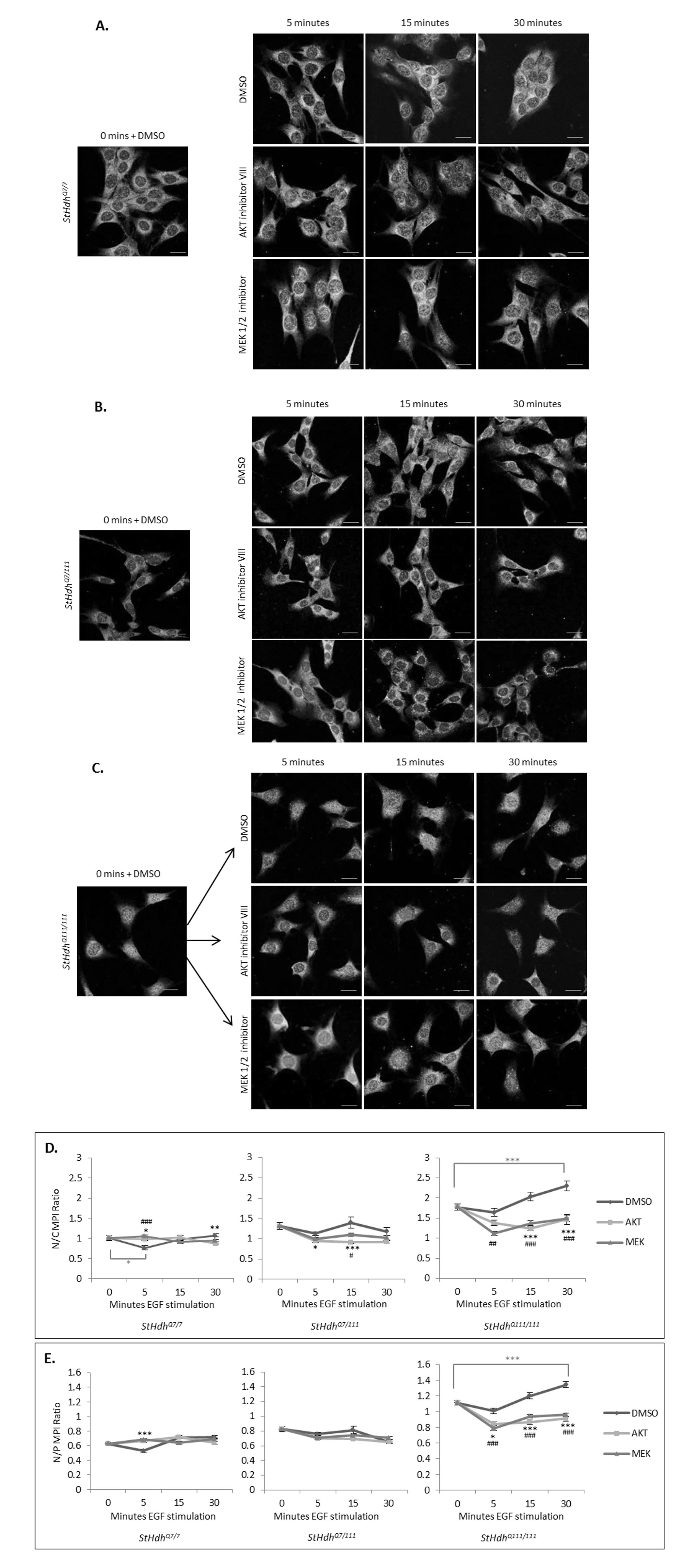

Supplement: S7 Fig — Error bars = SEM. Light grey bars and asterisks signify statistically significant differences between DMSO conditions. Black asterisks and hashes indicate statistically significant differences between DMSO vs AKT inhibitor conditions and DMSO vs MEK inhibitor conditions, respectively. Data representative of three experiments. n = 78–140. */# p<0.05, **/## p<0.01, ***/### p<0.001. (TIF) [file pone.0144864.s007.tif]

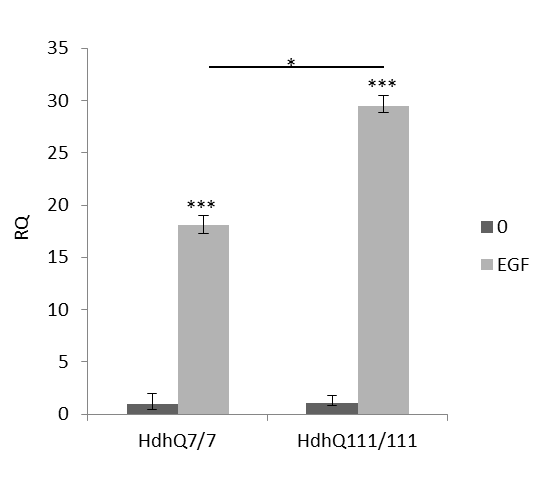

Supplement: S8 Fig — Statistical analysis was conducted on ΔCt values. Egr1 expression in both genotypes was significantly increased following EGF stimulation (both p<0.001), and the extent of this increase was significantly larger in HdhQ111/111 primary cells compared to HdhQ7/7 cells (p<0.05). Error bars = ± SEM. N = 5. * p<0.05, ** p<0.01, ***p<0.001. (TIF) [file pone.0144864.s008.tif]
